# Editorial: Improvement of Rice Through “-omics” Approaches

**DOI:** 10.3389/fpls.2022.926976

**Published:** 2022-05-24

**Authors:** Ravi Gupta, M. Iqbal R. Khan, Jose Luis Gonzalez Hernandez, Wei Wang, Laurence Veronique Bindschedler

**Affiliations:** ^1^College of General Education, Kookmin University, Seoul, South Korea; ^2^Department of Botany, Jamia Hamdard, New Delhi, India; ^3^Department of Agronomy, Horticulture, and Plant Science, South Dakota State University, Brookings, SD, United States; ^4^College of Life Sciences, Henan Agricultural University, Zhengzhou, China; ^5^Department of Biological Sciences, Royal Holloway University of London, Egham, United Kingdom

**Keywords:** rice, omics, yield, food security, stress

Rice is one of the most consumed food worldwide and is the first crop to have a complete genome sequence (Goff et al., 2002; Yu et al., 2002). However, rice productivity is at risk during this climate-changing era and thus improvement of rice is of utmost importance to address the issue of global food security. Advancements in omics technologies provided a platform for systemic dissection of regulatory pathways underlying rice development, growth, and productivity during optimal or stressful growth conditions. Consequently, tremendous genomics and transcriptomics studies were performed in the last few decades that identified hundreds of desirable genes and QTLs related to yield and stress tolerance (Song et al., 2018). More recently, this has been supported by multi-omics studies to further identify related genes, proteins, and metabolites (Khan et al., 2021).

## Strategies to Improve Proteome Coverage

Membrane proteomics has allowed the investigation of cell signaling pathways despite the associated challenges of working with highly hydrophobic proteins. To overcome such limitations, and increase the identification of key signaling pathways that may lead to crop improvement in terms of yield and tolerance to environmental stresses, Van Nguyen et al. developed an azo chemical to improve the solubilization of rice microsomal membrane proteins. Results obtained suggested a higher efficacy of azo in solubilizing the microsomal proteins than the well-known anionic surfactant SDS and suggested that the azo can be successfully utilized for the large-scale identification of membrane proteins.

## Trait Improvement and Yield Enhancement

Improvement of grain quality and yield is a key goal governing rice research as exemplified by the following papers published in the present issue. A mini-review by Ding et al. provides a short overview on cis-regulator elements (CREs) regulating gene expression in rice and how their targeting *via* genome editing techniques can be exploited to improve rice grain quality while increasing our understanding of gene expression regulatory networks. In the present issue, several studies focused on embryo development, and its impact on endosperm quality as these are determining features for size and grain quality in rice. The study by Hu et al. identified genes involved in the regulation of embryo size, such as the *GIANT EMBRYO* (*GE*) gene. The authors showed that a “giant embryo” phenotype was due to a single nucleotide deletion in the first exon of the *GE*. Furthermore, a comparison of the gene expression and metabolite accumulation in two cultivars differing in the size of their embryo suggested an increase in biosynthesis of L-Aspartic acid and L-Tryptophan in the giant embryo cultivar. The study by Tao et al. emphasized the role played by the embryo in the formation of the chalky endosperm, which is a key desired quality trait of the rice grain. Non-targeted metabolomics and NGS RNAseq were performed in a notched-belly (NB) mutant which is exhibiting a distinct separation between the translucent and chalky regions of the endosperm. Data showed that the embryo negatively regulates the accumulation of sucrose, amino acid, starch, and storage proteins in the chalky region of the endosperm by reducing the expression of starch, prolamin, and glutelin synthesis-related genes, suggesting that the embryo induces a metabolic shift in the endosperm which is important in developing high-quality rice by balancing embryo-endosperm interaction.

In another study, Andrew-Peter-Leon et al. focused on the identification of gene loci responsible for controlling yield, following the mutagenization of an Improved White Ponni (IWP) variety. One of the mutant lines, WP-22-2, exhibited semi-dwarfism, early flowering, and higher yield without alteration of grain quality. Whole-genome sequencing identified large-scale deletions and single nucleotide polymorphism (SNP), including mutations that lead to the functional disruption of *OsGA2oox2* and *OsFBX267*. These results suggest that both of these genes can be exploited for the development of early maturing and semi-dwarf varieties of rice. Bhatta et al. used and showed the efficacy of an image-based geometric traits phenotyping method for selecting rice genotypes tolerant to low phosphorus conditions.

## Molecular Signaling of Rice Under Stress Conditions

Plant growth and crop yield are severely constrained under stress conditions. Thus, understanding plant responses to stress are instrumental for engineering stress-tolerant rice cultivars. In this issue, two reviews describe multi-omics studies describing rice responses to drought (Zargar et al.) and other abiotic and biotic stresses (Iqbal et al.), to catalog the key components involved in trait management and yield enhancement under stress conditions. In an original, study, Chutimanukul et al. utilized an integrated genome and gene co-expression network analysis to identify candidate genes potentially involved in the salinity stress response in rice, such as *OsNodulin, OsBTBZ1, OsPSB28, OsERD, OsSub34, peroxidase precursor* genes, as well as investigating gene co-expression network (GCN) involved in the regulation of salinity stress response in rice. Likewise, in the study by Sonsungsan et al., transcriptome profiling of a salt-tolerant rice variety identified 107 salinity responsive genes related to the sensing of salt stress, signaling, hormone response, and gene regulation. Lee et al. investigated the effects of salinity stress on developing rice seeds using a RNASeq-based study comparing the transcriptome of the Samgwang rice cultivar cultivated in reclaimed and normal land. It was shown that this rice cultivar “Samgwang” showed higher sensitivity to salinity stress when cultivated in the reclaimed land, showing adverse effects such as decreased yield, grain weight, palatability, and amylose content, and this was associated with upregulation of genes related to the biosynthesis of ABA, trehalose, raffinose, and maltose under salinity stress conditions. Interestingly, Khan et al. reported that the impact of salinity stress in rice can be significantly reversed by the application of sugarcane press-mud. It was shown that the application of press mud induced the activity of antioxidant enzymes and promoted the growth, yield, total soluble proteins, free amino acids, and soluble sugars, thus mitigating the negative effects of salinity stress. Similarly, Huang et al. reported that biochar application can protect rice against heat-induced damage possibly *via* alteration of the soil properties and its microbiome in the root zone environment. Decreased heat damage following biochar treatment was associated with decreased abundance of heat stress markers such as heat-shock proteins.

Altogether, the review articles and original studies reported in this Research Topic are contributing to the general understanding of the molecular mechanisms regulating rice development and responses to environmental stresses, in particular for their impact on grain quality and crop yield and such information will be instrumental for engineering new rice varieties, with improved grain quality, yield, and stress tolerance.

## Author Contributions

All authors listed have made a substantial, direct, and intellectual contribution to the work and approved it for publication.

## Conflict of Interest

The authors declare that the research was conducted in the absence of any commercial or financial relationships that could be construed as a potential conflict of interest.

## Publisher's Note

All claims expressed in this article are solely those of the authors and do not necessarily represent those of their affiliated organizations, or those of the publisher, the editors and the reviewers. Any product that may be evaluated in this article, or claim that may be made by its manufacturer, is not guaranteed or endorsed by the publisher.
